# Risk of progression in patients with chronic atrophic gastritis: A retrospective study

**DOI:** 10.3389/fonc.2022.942091

**Published:** 2022-08-01

**Authors:** Lu Sun, Xiaoliang Jin, Liang Huang, Jing Zhao, Haifeng Jin, Mingtao Chen, Chunli Zhang, Bin Lu

**Affiliations:** ^1^ Department of Gastroenterology, First Affiliated Hospital of Zhejiang Chinese Medical University, Hangzhou, China; ^2^ Department of Endoscopy Center, First Affiliated Hospital of Zhejiang Chinese Medical University, Hangzhou, China; ^3^ Department of Pathology, First Affiliated Hospital of Zhejiang Chinese Medical University, Hangzhou, China; ^4^ Key Laboratory of Digestive Pathophysiology of Zhejiang Province, First Affiliated Hospital of Zhejiang Chinese Medical University, Hangzhou, China

**Keywords:** gastric cancer, early detection of cancer, follow-up studies, atrophic gastritis, endoscopy

## Abstract

**Background:**

Chronic atrophic gastritis (CAG) can progress to gastric cancer (GC) thus requiring endoscopic surveillance. Here, we analyze various aspects of CAG progression, time, and mucosal background, to guide reasonable surveillance.

**Methods:**

CAG patients with three or more endoscopies from 2010–2021 were included. All cases were analyzed for rate and time of progression, and cases with operative link on gastritis assessment (OLGA) staging, operative link on gastric intestinal metaplasia assessment (OLGIM) staging, and Kimura-Takemoto classification were further analyzed. Additional investigation of guideline-defined low-risk patients by reviewing endoscopy in the short-term (1–2 years) after baseline identified several patients as high-risk.

**Results:**

Ninety-seven (10.4%) of the 929 CAG patients progressed to low-grade intraepithelial neoplasia (LGIN), high-grade intraepithelial neoplasia (HGIN), or GC, during the observation period of 36–129 months (median 53, IQR=24), including 75 (8.1%) cases of LGIN, eight (0.9%) of HGIN, and 14 (1.5%) of GC. Among 170 patients with OLGA/OLGIM at baseline, two (2/2, 100%) GC cases occurred in patients with OLGA/OLGIM III and IV. Of the 236 patients with Kimura-Takemoto classification at baseline, 5/7 (71.4%) cases of GC occurred in patients with C3–O3. Ten, 11, and 25 patients classified as low-risk on the European, British, and Chinese Guidelines, underwent additional endoscopy within 1–2 years, resulting in three (30.0%), four (36.4%), and eight (32.0%) patients being classified as high-risk on these guidelines, respectively.

**Conclusion:**

A minority of CAG patients can progress to GC. OLGA/OLGIM III and IV staging are closely associated with progression. Disease-associated risk may be underestimated in one-third of patients classified as low-risk by initial endoscopy.

## Introduction

Gastric cancer (GC) is one of the five most common types of cancer, and the third most deadly malignancy worldwide (1). However, if early gastric cancer (EGC) is detected on time and removed *via* endoscopy or surgery, the 5-year survival rate of patients can be increased to more than 90% (2). Therefore, early diagnosis of GC is crucial for improving prognosis. Chronic atrophic gastritis (CAG) is a gastric epithelial precancerous condition and has a risk of progression to GC. A long-term follow-up study in the Netherlands (3) showed that the annual incidence of gastric cancer in patients with CAG was 0.1%–0.25%, while another study showed a rate of 0.3% (4). In addition, the annual incidence of neoplastic lesions (including intraepithelial neoplasia and GC) in CAG patients can be as high as 1.36% (5). Moreover, a long-term follow-up study demonstrated that about 1/50 of gastric atrophy (GA) and 1/39 of intestinal metaplasia (IM) patients eventually progress to GC within 20 years (6). Therefore, scheduling endoscopic surveillance of patients with CAG can help detect EGC in a timely manner, thereby improving the survival rate of patients with GC. The risk of CAG progression to GC is closely related to the extent and degree of GA and IM (7, 8). Hence, the risk of cancer in CAG should be stratified in order to make different endoscopic surveillance plans. Currently, international and national guidelines or consensus on endoscopic surveillance of gastric epithelial precancerous conditions and lesions propose endoscopic surveillance of CAG patients according to different risk stratification methods.

This study aimed to analyze the risk of development of GC in CAG patients, the relationship between lesion progression and gastric mucosal background, and the time course of lesion progression, in order to provide a basis for a reasonable clinical endoscopic surveillance plan.

## Materials and methods

### Patient selection

This was a retrospective study that enrolled patients who underwent endoscopy at our endoscopy center from January 2010 to November 2021, and who had CAG confirmed by pathology. The inclusion criteria were: 1) age ≥18 years, 2) number of endoscopic surveillances ≥3 times, 3) at least 3 years between the time of the last and baseline endoscopy. The exclusion criteria were: 1) history of upper gastrointestinal surgery, 2) history of gastric neoplasms or other unremitting neoplasms, 3) baseline endoscopic pathological biopsy confirmed existing intraepithelial neoplasia or gastric cancer. The study was approved by the ethical review committee of the First Affiliated Hospital of Zhejiang Chinese Medical University (2021-KL-206-01).

### Data collection

We collected the demographic information and endoscopic and pathological data of CAG patients, including basic information such as sex, age, and date of examination, as well as biopsy site, number of biopsies, pathological diagnoses, degree of lesion, and *Helicobacter pylori* (*H. pylori*) infection status. Endoscopic photographs, endoscopic diagnosis, lesion extent, lesion site, and Kimura-Takemoto classification, were also collected. OLGA and OLGIM staging were performed for cases in which five biopsies were performed, according to the updated Sydney System.

### Research methods

**Figure 1:**
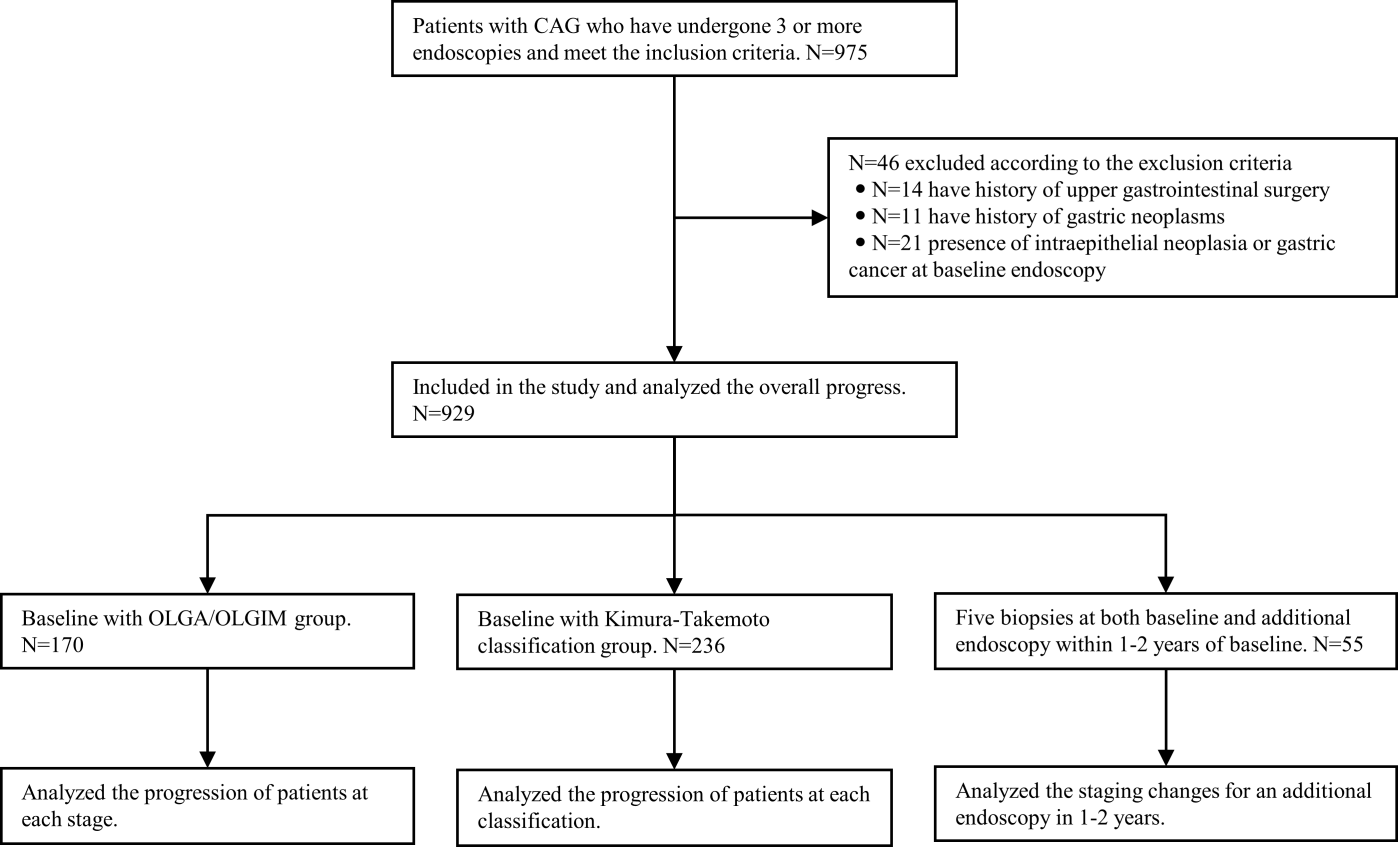
Flowchart of the study design. CAG, chronic atrophic gastritis; OLGA, operative link on gastritis assessment; OLGIM, Operative link on gastric intestinal metaplasia assessment.

1. The changes in lesions during the follow-up period were analyzed, and were defined as progression, stable, or reversal. Progression was defined as the development of LGIN, HGIN or GC in CAG patients during the follow-up period. Stable was defined as the persistence of GA and IM in CAG patients during the follow-up period. Reversal was defined as the disappearance of GA and/or IM in CAG patients during the follow-up period.
2. OLGA and OLGIM staging were performed for cases with five-site biopsies at the baseline endoscopy, according to the updated Sydney System (9). Lesion progression and gastric carcinogenesis at each stage were analyzed at follow-up.
3. Cases in which gastritis was classified according to the Kimura-Takemoto classification at baseline endoscopy were analyzed for lesion progression and gastric carcinogenesis at each stage during follow-up.
4. Cases with antrum and corpus biopsies and complete pathological data during endoscopic surveillance were divided into low and high-risk groups, according to the management of epithelial precancerous conditions and lesions in the stomach (MAPS II) (10), the British Society of Gastroenterology guidelines on the diagnosis and management of patients at risk of gastric adenocarcinoma (BSG) (11), and the Chinese consensus on the management of gastric epithelial precancerous conditions and lesions (12). In principle, MAPS II and BSG define patients with CAG whose lesions are confined to the antrum as low-risk, and patients whose lesions involve the antrum and corpus, as high-risk. Low-risk patients do not require endoscopic surveillance, while high-risk patients require endoscopy every 3 years. The Chinese consensus defines patients with mild-to-moderate GA and IM as low-risk, requiring endoscopy every 3 or 2–3 years, and those with severe GA and IM as high-risk, requiring surveillance every 1–2 years. Among patients in the low-risk group defined by different guidelines, the proportion defined as high-risk was analyzed for reassessment at endoscopy within 1–2 years after the baseline (**Figure 1**).

### Statistical analysis

All analyses were performed using the SPSS v25.0 software. Normally distributed data were expressed as mean with standard deviation (SD), skewed data were expressed as median with interquartile range (IQR), and rates and composition ratios were expressed as N (%). A chi-square test and/or Fisher’s exact test was used to compare differences in the risk of progression in patients with different mucosal staging. This included differences in the risk of progression between OLGA 0–II and OLGA III–IV, OLGIM0–II and OLGIM III–IV, and Kimura–Takemoto C1–C2 and C3–O3; *p* < 0.05 indicated statistical significance. Moreover, the odds ratio (OR) was used to reflect the effect of high mucosal staging on the role of the risk of progression.

## Results

**Figure 2:**
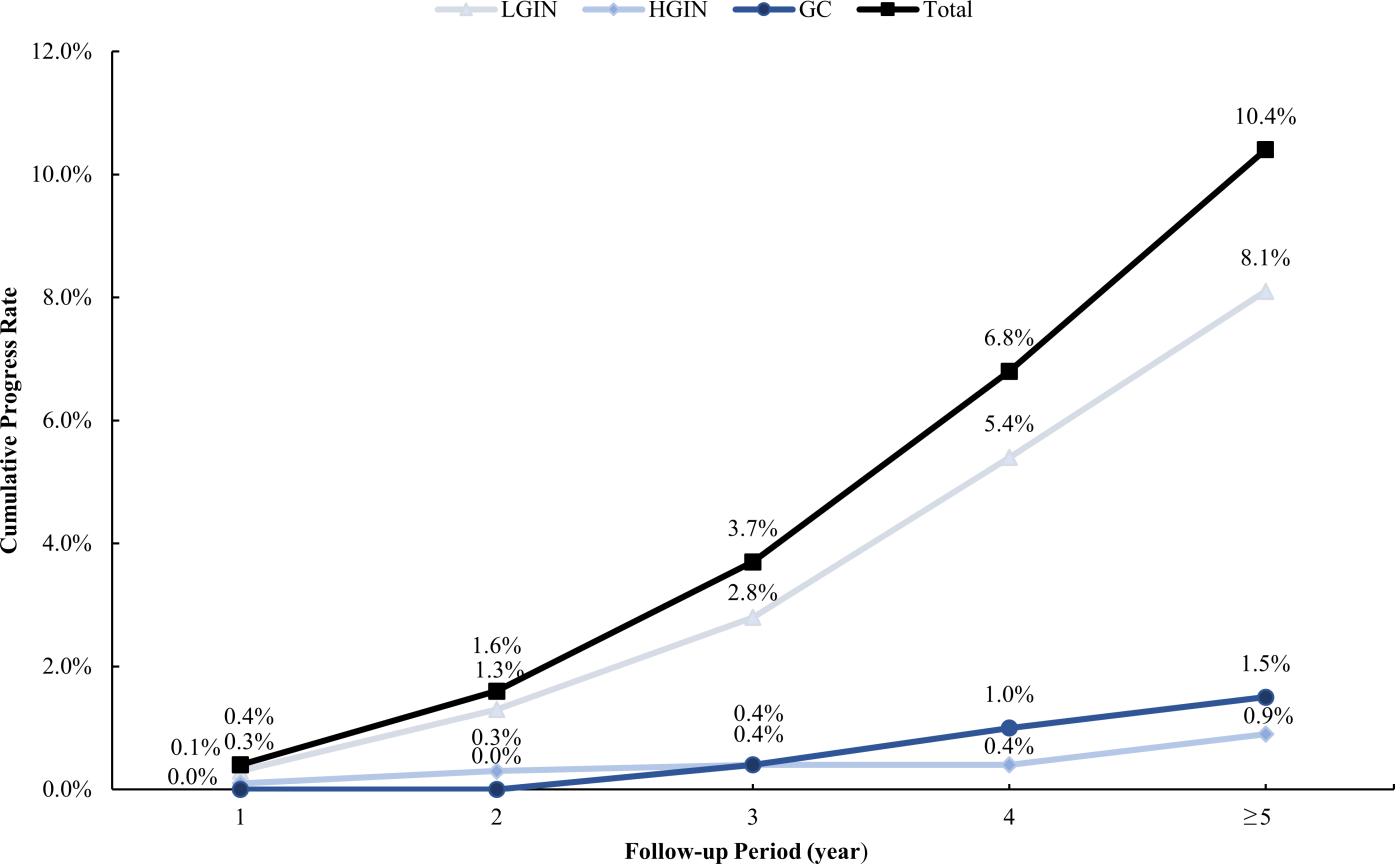
Cumulative rate of progress during the follow-up period. LGIN, low-grade intraepithelial neoplasia; HGIN, high-grade intraepithelial neoplasia; GC, gastric cancer.

**Figure 3:**
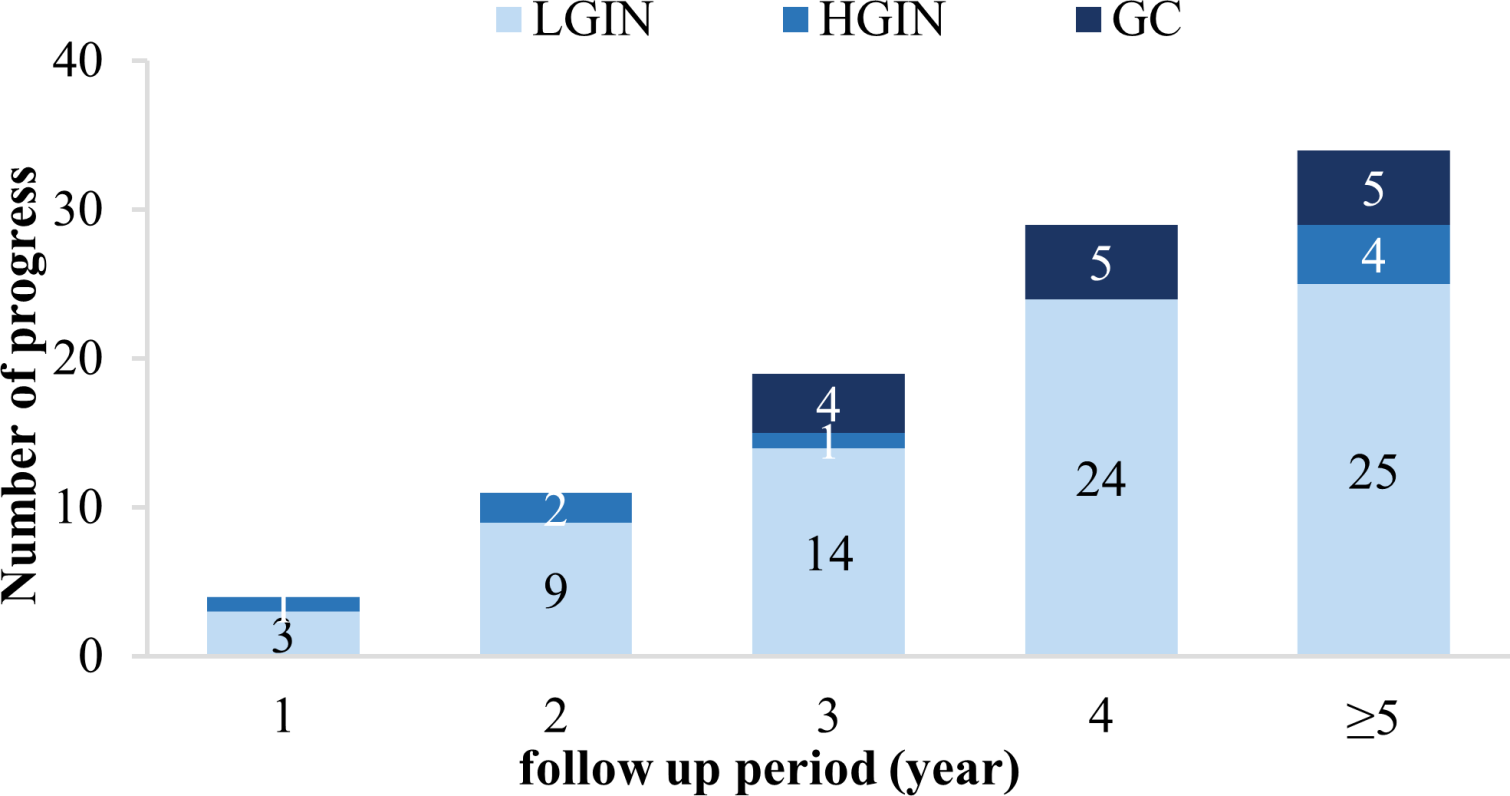
Progression of the included patients during each year of the follow-up period. LGIN, low-grade intraepithelial neoplasia; HGIN, high-grade intraepithelial neoplasia; GC, gastric cancer.

Our study enrolled 929 CAG patients. Their age range was 32–80 (mean 56.59 ± 10.85) years, and 48.9% were men, as described in **Table 1**. During the 36–129-month follow-up period (median 53, IQR=24), 97 cases (10.4%) progressed to IN or GC, of which 75 (8.1%) were LGIN, 8 (0.9%) were HGIN, and 14 (1.5%) were GC. The relationship between lesion progression and follow-up time is indicated in **Figures 2**, **3**. The results revealed that the proportion of lesion progression increased with time, with GC cases overall being detected at year 3 and beyond, and the majority of HGIN cases (7/8, 87.5%) being detected at year 2 and beyond, during the follow-up period.

**Table 1:** Baseline characteristics of the included patients.

All included patients	Total number of people (n=929)
Sex (male, %)	48.9
Age (Mean ± SD)	56.59 ± 10.85
Number of months of observation (Median, IQR)	53 (24)
*Helicobacter pylori* infection	288 (31.0%)
OLGA group	Number of OLGA group (n=170)
OLGA 0	8 (4.7%)
OLGA I	84 (49.4%)
OLGA II	42 (24.7%)
OLGA III	23 (13.5%)
OLGA IV	13 (7.6%)
OLGIM group	Number of OLGIM group (n=170)
OLGIM 0	2 (1.2%)
OLGIM I	72 (42.4%)
OLGIM II	49 (28.8%)
OLGIM III	31 (18.2%)
OLGIM IV	16 (9.4%)
Kimura-Takemoto group	Number of Kimura-Takemoto group (n=236)
C-1	42 (17.8%)
C-2	111 (47.0%)
C-3	53 (22.5%)
O-1	13 (5.5%)
O-2	15 (6.4%)
O-3	2 (0.8%)

SD, standard deviation; IQR, interquartile range; OLGA, operative link on gastritis assessment; OLGIM, Operative link on gastric intestinal metaplasia assessment.

Altogether, 641 patients (69.0%) were negative for *H. pylori* at baseline, or had previously eradicated *H. pylori*, and 5 cases (0.8%) eventually progressed to GC. *H. pylori* infection was present in 288 (31.0%) patients at baseline. These patients were treated, and eventually *H. pylori* was eradicated in 246 cases during the follow-up period; two of these cases (0.7%) progressed to GC. *H. pylori* eradication failed in 42 cases, and 7 (16.7%) of them progressed to GC.

Seventy-five cases of LGIN were detected, of which 54 (72.0%) were endoscopically visualized lesions obtained by targeted biopsy and 21 (28.0%) lacked visualized lesions and were obtained by random biopsy. Visual lesions mainly exhibited rough mucosal erosions in 18 cases (24.0%), hyperplastic bulges in 17 cases (22.7%), mucosal redness in 13 cases (17.3%), and ulcerative lesions in six cases (12.0%). Ten cases (13.3%) of LGIN underwent endoscopic submucosal dissection (ESD) resection once detected, whereas the rest of the lesions were managed under observation. At the follow-up, 37 (49.3%) lesions regressed: 16 non-visible lesions regressed pathologically, 13 of 21 visible lesions regressed both pathologically and endoscopically and another 8 regressed pathologically but were still visible endoscopically, 3 cases (4.0%) had maintained lesions, and 4 (5.3%) progressed (including 3 of GC and 1 of HGIN, all of which were resected with ESD after progression). Another 21 cases (28.0%) were found at the time of the last endoscopy and therefore no follow-up was recorded at this time.

Eight cases of HGIN were detected, all of which were endoscopically visualized lesions obtained by targeted biopsy. The main endoscopic findings were redness of the mucosa in three cases (37.5%), rough mucosal erosion in two cases (25.0%), hyperplastic bulge in two cases (25.0%), and ulcerative lesion in one case (12.5%). All these lesions were excised with ESD after detection.

Fourteen patients eventually progressed to GC, most of which were detected after year 3 (four cases in year 3; five in year 4; one each in years 5, 6, and 7; and two in year 10). Ten cases were well-differentiated adenocarcinoma, three were moderately differentiated adenocarcinoma, and one was poorly moderately differentiated adenocarcinoma. One case of poorly differentiated adenocarcinoma had invaded the serosa and was finally removed by surgery. The remaining 13 cases of well and moderately differentiated adenocarcinomas were EGC, 11 of which invaded the lamina propria, one reached the muscularis mucosa, and one reached the submucosa; all these EGC lesions were resected *via* ESD. The information of the 14 patients who progressed to GC is detailed in **Table 2**.

**Table 2:** Information on cases that progressed to gastric cancer during the follow-up period.

No.	Age	Sex	Endoscopic features at baseline				Time from baseline (months)	Treatment	Pathology of GC		
			Date	Kimura-Takemoto	GA	IM			Level of differentiation	Histology	Depth
1	60	Female	2016.07	C2^a^	Mild (antrum)	Mild (antrum)	39	ESD	moderate	adenocarcinoma	lamina propria
2	69	Female	2014.11	superficial	OLGA I^b^	OLGIM I^b^	78	ESD	moderate	adenocarcinoma	lamina propria
3	56	Male	2019.05	C3	OLGA III^b^	OLGIM III^b^	25	ESD	well	adenocarcinoma	lamina propria
4	45	Male	2016.11	C2	OLGA II^b^	OLGIM I^b^	43	ESD	well	adenocarcinoma	lamina propria
5	67	Male	2017.05	O2^a^	OLGA I^b^	OLGIM II^b^	35	ESD	well	adenocarcinoma	lamina propria
6	63	Male	2013.08	C2	Mild (antrum)	Mild (antrum)	59	ESD	well	adenocarcinoma	lamina propria
7	77	Male	2015.01	C2^a^	OLGA III	OLGIM III	35	ESD	well	adenocarcinoma	lamina propria
8	50	Female	2017.05	O3	OLGA III^b^	OLGIM III^b^	48	ESD	well	adenocarcinoma	lamina propria
9	64	Male	2010.11	C3	Mild (antrum)	Severe (antrum)	120	ESD	well	adenocarcinoma	lamina propria
10	59	Female	2011.10	C2^a^	Severe (antrum)	Mild (antrum)	114	ESD	well	adenocarcinoma	lamina propria
11	63	Female	2013.06	O2^a^	OLGA III^b^	OLGIM III^b^	72	ESD	moderate	adenocarcinoma	submucosa
12	64	Male	2017.03	C3	OLGA III^b^	OLGIM III^b^	48	ESD	well	adenocarcinoma	muscularismucosa
13	67	Male	2017.08	O2	OLGA IV	OLGIM IV	30	Surgery	poor	adenocarcinoma	serosa
14	56	Male	2018.05	superficial	None (antrum)	Mild (antrum)	41	ESD	well	adenocarcinoma	lamina propria

a The endoscopic data had a lack of pictures or text descriptions, and the Kimura-Takemoto classification was eventually determined by picture rereading, text description analysis, or telephone follow-up.

b Pathological biopsy sampling included the gastric sinus and body but could not attain the five pieces required according to the updated Sydney System, so the OLGA/OLGIM judged in this way may have down-staged, or underestimated the disease.

OLGA, operative link on gastritis assessment; OLGIM, operative link on gastric intestinal metaplasia assessment; GA, gastric atrophy; IM, intestinal metaplasia; GC, gastric cancer; ESD, endoscopic submucosal dissection.

**Figure 4:**
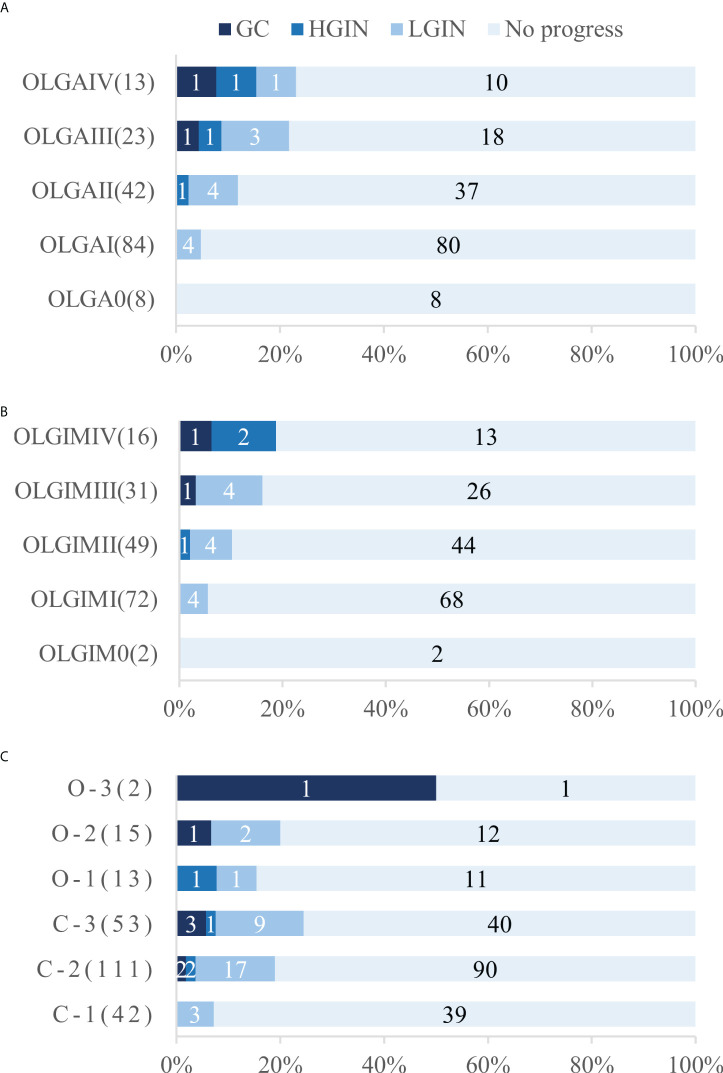
Number and proportion of patients who underwent progression in each stage and classification. **(A)** Number and proportion of patients who underwent progression in each OLGA stage **(B)** Number and proportion of patients who underwent progression in each OLGIM stage **(C)** Number and proportion of patients who underwent progression in each Kimura-Takemoto classification. OLGA, operative link on gastritis assessment; OLGIM, Operative link on gastric intestinal metaplasia assessment; LGIN, low-grade intraepithelial neoplasia; HGIN, high-grade intraepithelial neoplasia; GC gastric cancer.

A total of 170 patients (47.1% men, mean age 57.1) underwent standard 5-site biopsy at baseline endoscopy according to the updated Sydney System. At the 36–120-month follow-up (median 52), a total of 17 cases (10.0%) progressed to IN or GC, including 12 cases (7.1%) progressing to LGIN, 3 (1.8%) progressing to HGIN, and 2 (1.2%) progressing to GC. The number of progressions, progress rate, and progress time for each stage are detailed in **Table 3** and **Figure 4**. The results show that two cases (2/2, 100%) of GC and two cases (2/3, 66.7%) of HGIN occurred in the background with baseline OLGA/OLGIM III and IV. Both GC cases occurred in year 3. The overall progression rate for OLGA III–IV and OLGA0–II was 22.2% and 6.7%, respectively, *p* = 0.015, OR=3.309 (95% CI 1.375-7.964). The rates of HGIN and GC progression in OLGA III–IV and OLGA0–II were 11.1% and 0.7%, respectively, *p* = 0.007, OR = 14.899 (95% CI 1.717-129.129). The overall progression rates of OLGIM III–IV and OLGIM0–II were 17.0% and 7.3%, respectively, *p* = 0.105, OR = 2.326 (95% CI 0.954-5.671). The progression rates of HGIN and GC of OLGIM III–IV and OLGIM0–II were 8.5% and 0.8%, respectively, *p* = 0.032, OR = 10.468 (95% CI 1.201-91.260). GC and HGIN were eventually resected *via* ESD or surgery.

**Table 3:** Lesion progression in the updated Sydney System group and Kimura-Takemoto classification group.

Staging/Classification	Lesions			*P*/OR	Time distribution of each lesion progression(year)				
	LGIN	HGIN	GC		1	2	3	4	≥5
OLGA0 (8)	0 (0.0%)	0 (0.0%)	0 (0.0%)	*P1 =* 0.015 OR1 = 3.309 *P2 =* 0.007 OR2 = 14.899					
OLGAI (84)	4 (4.8%)	0 (0.0%)	0 (0.0%)		LGIN*1			LGIN*2	LGIN*1
OLGAII (42)	4 (9.5%)	1 (2.4%)	0 (0.0%)			LGIN*2		LGIN*2	HGIN*1
OLGAIII (23)	3 (13.0%)	1 (4.3%)	1 (4.3%)		LGIN*2	LGIN*1	HGIN*1+GC*1		
OLGAIV (13)	1 (7.7%)	1 (7.7%)	1 (7.7%)		HGIN*1	LGIN*1	GC*1		
OLGIM0 (2)	0 (0.0%)	0 (0.0%)	0 (0.0%)	*P3 =* 0.105 OR3 = 2.326 *P4 =* 0.032 OR4 = 10.468					
OLGIMI (72)	4 (5.6%)	0 (0.0%)	0 (0.0%)		LGIN*1			LGIN*2	LGIN*1
OLGIMII (49)	4 (8.2%)	1 (2.0%)	0 (0.0%)		LGIN*1	LGIN*1		LGIN*2	HGIN*1
OLGIMIII (31)	4 (12.9%)	0 (0.0%)	1 (3.2%)		LGIN*1	LGIN*3	GC*1		
OLGIMIV (16)	0 (0.0%)	2 (12.5%)	1 (6.3%)		HGIN*1		HGIN*1+GC*1		
C-1 (42)	3 (7.1%)	0 (0.0%)	0 (0.0%)	*P5 =* 0.171 OR5 = 1.459 *P6 =* 0.089 OR6 = 3.226			LGIN*2	LGIN*1	
C-2 (111)	17 (15.3%)	2 (1.8%)	2 (1.8%)			LGIN*2	LGIN*6	LGIN*6+GC*1	LGIN*3+HGIN*2+GC*1
C-3 (53)	9 (17.0%)	1 (1.9%)	3 (5.7%)		LGIN*1	LGIN*1	GC*1	LGIN*4+GC*1	LGIN*3+HGIN*1+GC*1
O-1 (13)	0 (0.0%)	1 (7.7%)	1 (7.7%)		HGIN*1	LGIN*1			
O-2 (15)	2 (13.3%)	0 (.0%)	1 (6.7%)		LGIN*1		GC*1	LGIN*1	
O-3 (2)	0 (0.0%)	0 (0.0%)	1 (50.0%)					GC*1	

*P1, OR1*: P value and odds ratio in overall progression rates between OLGA III-IV and OLGA 0-II. *P2, OR2*: P value and odds ratio in HGIN, GC progression rates between OLGA III-IV and OLGA0-II. *P3, OR3*: P value and odds ratio in overall progression rates between OLGA III-IV and OLGA0-II. *P4, OR4*: P value and odds ratio in HGIN, GC progression rates between OLGA III-IV and OLGA0-II. *P5, OR5*: P value and odds ratio in overall progression rates between C3-O3 and C1-C2. *P6, OR6*: P value and odds ratio in HGIN, GC progression rates between C3-O3 and C1-C2.

OLGA, operative link on gastritis assessment; OLGIM, Operative link on gastric intestinal metaplasia assessment; LGIN, low-grade intraepithelial neoplasia; HGIN, high-grade intraepithelial neoplasia; GC, gastric cancer. * means multiplication sign (x).

Overall, 236 patients (47.9% men, mean age 56.9) were classified according to the Kimura-Takemoto classification at baseline endoscopy. At the 36–120-month follow-up (median 50), a total of 43 cases (18.2%) progressed to IN or GC, including 32 (13.6%) to LGIN, 4 (1.7%) to HGIN, and 7 (3.0%) to GC. The number of progressions, progression rate, and progression time for each classification are detailed in **Table 3** and **Figure 4**. The results reveal that GC mainly occurred in patients with C–3 to O–3 (5/7, 71.4%), and was detected at the 3-year follow-up and beyond. Moreover, two (2/4, 50%) cases of HGIN occurred at C–3 to O–3. The overall progression rates for the Kimura-Takemoto classification C3–O3 and C1–C2 were 22.9% and 15.7%, respectively, *p* = 0.171, OR = 1.459 (95% CI 0.851-2.502). The progression rate of HGIN and GC in C3–O3 versus C1–C2 was 8.4% versus 2.6%, respectively, *p* = 0.089, OR = 3.226 (95% CI 0.973-10.700). GC and HGIN were eventually resected *via* ESD or surgery.

Fifty-five patients underwent biopsies of the antrum and corpus during endoscopic surveillance throughout the follow-up period. According to the MAPS II guideline, 18 and 37 cases were classified as low and high risk, respectively. An additional 10 cases in the low-risk group underwent an additional endoscopy within 1–2 years. We found that three cases (30.0%) were defined as high risk on the subsequent endoscopy, meaning that there may be a 30.0% miscalculation in assessing the risk of progression in patients with CAG with only one endoscopy.

Similarly, 19 cases in the low-risk group and 36 in the high-risk group were identified according to the BSG guideline. Conversely, 11 cases in the low-risk group had an additional endoscopy within 1–2 years and four (36.4%) were classified as high-risk, meaning that the risk of progression in patients with CAG assessed by only one endoscopy may be misclassified in 36.4% of cases.

According to the Chinese consensus, 39 and 16 cases were classified as low and high risk, respectively. Among the low-risk group, 25 cases underwent an additional endoscopy within 1–2 years, and 8 (32.0%) were identified as high-risk, meaning that the risk of progression in patients with CAG assessed by only one endoscopy was likely to be misclassified in 32.0% of cases.

## Discussion

Many studies have demonstrated that eradication of *H. pylori* infection can reduce or even reverse GA, especially in early or mild atrophy (13). However, the reversal of severe GA as well as IM is difficult (14). Several recent studies with longer follow-up periods have shown that IM can also be gradually reduced after longer periods of *H. pylori* eradication (15), although reversal of IM is slower than GA and may only become apparent 5 years after *H. pylori* eradication. In the remaining CAG patients, the majority of the lesions remained largely stable, while a small number progressed. Appropriate endoscopic surveillance of patients with CAG allows for early detection of progression or cancer risk and timely resection of the lesion, thereby improving patient survival. It has previously been shown that endoscopic surveillance of CAG patients once every 1–3 years is cost-effective (16, 17). Although these studies are from different countries and regions, countries can develop different surveillance schemes for different patients, considering the risk of progression of CAG and other national conditions.

Ninety-seven (10.4%) of the 929 CAG patients in the current study progressed to IN or GC during a follow-up period of 36–129 months (median 53), including 75 cases (8.1%) of LGIN, 8 cases (0.9%) of HGIN, and 14 cases (1.5%) of GC. Progression to GC occurred mainly after year 3 of the follow-up period, while HGIN was found predominantly after year 2. In comparison to that of previous studies with larger samples (18), GC occurred mostly in CAG patients with 3–4 years of follow-up and beyond, although individual GC also occurred in years 1–2, which is similar to the results of the present study. IN is a precancerous lesion that can occur as early as 1–2 years into the follow-up period of CAG patients (4). HGIN has a high risk of progressing to GC whereas LGIN has a low risk (19). In addition, we further followed up some of the LGIN cases and found that most LGIN lesions eventually regressed, with only a few progressing. Previous long-term follow-up studies on LGIN showed that 38%–75% of LGIN regressed spontaneously, 19%–50% persisted, and 0–23% became cancerous (20), with an annual cancer rate of 0.6% (3). In comparison, the persistence rate was not as high in the present study, which may be related to the fact that some of the severe LGIN lesions were resected earlier with ESD. Overall, the majority of patients with CAG had stable disease, with progression and reversal observed in a small number of patients during the long-term follow-up period. The OLGA/OLGIM staging method for CAG combines the mucosal pathology of the antrum and corpus to assess the extent and degree of GA/IM and can reliably predict the risk of progression to tumors in patients with different CAG. Prospective studies by Rugge et al. (8) and Lahner et al. (5) showed significantly higher cancer rates in patients with OLGA/OLGIM III and IV. Moreover, OLGA III/IV, OLGIM III/IV, and endoscopic moderate to severe GA and IM, are more common in GC patients than in non-GC patients (21, 22). Of the two cases of GC observed in this study, one occurred in a patient with baseline gastric mucosal OLGA/OLGIM IV, and the other patient had OLGA/OLGIM III. Two of the three patients (66.7%) who progressed to HGIN also had OLGA/OLGIM III and IV at baseline. This confirms that OLGA/OLGIM III and IV are closely related to GC progression and should be intensively followed up endoscopically.

Regular endoscopic surveillance of CAG patients is an important initiative for disease management and an effective method for detecting EGC. Since CAG patients have different cancer risks, they should be risk-stratified to allow individualized surveillance plans to be devised. MAPS II (10) considered patients with mild-to-moderate GA limited to the antrum without IM, and IM limited to the antrum or corpus without risk factors (family history of GC, autoimmune gastritis, and persistent *H. pylori* infection) as low-risk and not requiring surveillance. High-risk patients were defined as those with IM confined to the antrum or corpus with risk factors, moderate to severe GA, as well as IM involving both the antrum and corpus; such patients require endoscopic surveillance once every 3 years. The BSG (9) states that patients with GA or IM limited to the antrum and without risk factors (family history of GC, persistent *H. pylori* infection, etc.) are at low risk and endoscopic surveillance is not recommended. Patients with GA or IM involving both antrum and corpus, or with GA or IM limited to the antrum but with risk factors, are considered high-risk and require endoscopic surveillance once every 3 years. The Chinese consensus (12) recommends endoscopy every 3 years for patients with mild-to-moderate CAG with GA limited to the antrum, or every 2–3 years if accompanied by IM; these patients are defined as low risk. Patients with severe CAG (OLGIM stage III/IV) are classified as high risk and require endoscopy every 1–2 years. We selected low-risk cases with complete biopsies of the antrum and corpus according to the MAPS II and BSG criteria, analyzed their results at 1–2 years of re-endoscopy and pathology, and found that about 1/3 cases were classified as high-risk at re-evaluation within 1–2 years after baseline. This indicates that these cases may be underestimated as low-risk and lose the opportunity of regular endoscopic surveillance or even early detection of GC, if they follow the MAPS II and BSG guidelines. In contrast, surveillance every 2–3 years, as recommended by the Chinese consensus, is more in line with the reality of the high incidence of GC in China. Meanwhile, this study also suggests that about 1/3 of low-risk patients judged by initial endoscopy may be misdiagnosed; performing an additional gastroscopy after 1–2 years can help correct this misdiagnosis and accurately evaluate the risk of progression in CAG patients.

This study had several limitations that need to be acknowledged. First, it was a retrospective study with some shortcomings in terms of data. Moreover, only a small number of cases in which five biopsies were taken at first endoscopy according to the Sydney criteria were analyzed. Furthermore, in some cases the determination of the degree and extent of GA and IM was inaccurate. However, our limited data indicated that the risk of progression to GC in CAG patients is positively correlated with OLGA/OLGIM staging and Kimura-Takemoto classification. Second, the number of patients with five biopsies taken during the whole endoscopic surveillance was very small, which influences the accuracy of risk re-evaluation during the long-term surveillance. In clinical practice, five biopsies were limited by large extent of damage, high incidence of bleeding and other biopsy-related adverse reactions, and poor operability. Thus, it will be worthwhile to further explore how to simplify the biopsy process, in order to meet the needs of the re-evaluation, as closely as possible. For instance, there is evidence that random biopsies of three pieces of tissue from the lesser curvature of antrum, the angulus, and the lesser curvature of corpus, have 94% concordance in OLGA/OLGIM staging compared to that of the standard five biopsies (23). This smaller number of biopsies may facilitate the clinical operation. Furthermore, combining endoscopic devices may also be a viable approach; for example, narrow band imaging (NBI) has a high accuracy for IM (24), with a sensitivity of more than 90%; therefore, NBI has been recommended to guide targeted biopsies (25). In this manner, missed diagnoses due to random biopsies can be avoided. Third, certain clinical information, such as the detailed treatment of *H. pylori*, was incomplete in this study; this may have affected the CAG progression outcomes.

## Conclusions

In conclusion, the majority (70.6%) of CAG patients reported in this study remained stable during the long-term follow-up. A minority (1.5%) of CAG patients progressed to GC, with most cases of GC occurring after 3 years of follow-up. OLGA/OLGIM III and IV were closely associated with progression to GC. Finally, approximately 1/3 of patients judged as low-risk by initial endoscopy may have underestimated disease, and an additional endoscopy after 1–2 years can help correct this misjudgment.

## Data availability statement

The original contributions presented in the study are included in the article/supplementary material. Further inquiries can be directed to the corresponding author.

## Ethics statement

The studies involving human participants were reviewed and approved by the ethical review committee of the First Affiliated Hospital of Zhejiang Chinese Medical University. Written informed consent for participation was not required for this study in accordance with the national legislation and the institutional requirements

## Author contributions

BL designed and supervised the study including all data collection and analysis; LS performed most of the investigation, including data collection and analysis, and wrote the manuscript; XJ and LH assisted with the data collection and analysis; JZ, HJ, MC, and CZ assisted with the data collection. All authors have read and approved the manuscript.

## Funding

This study was supported by Collaboration program of Chinese traditional and Modern Medicine in Gastric Cancer, and National Natural Science Foundation of China (No. 81970470).

## Acknowledgments

We would like to acknowledge the reviewers for their precious comments on our study.

## Conflict of interest

The authors declare that the research was conducted in the absence of any commercial or financial relationships that could be construed as a potential conflict of interest.

## Publisher’s note

All claims expressed in this article are solely those of the authors and do not necessarily represent those of their affiliated organizations, or those of the publisher, the editors and the reviewers. Any product that may be evaluated in this article, or claim that may be made by its manufacturer, is not guaranteed or endorsed by the publisher.
